# Reduced Water Negatively Impacts Social Bee Survival and Productivity Via Shifts in Floral Nutrition

**DOI:** 10.1093/jisesa/ieaa114

**Published:** 2020-10-06

**Authors:** Erin E Wilson Rankin, Sarah K Barney, Giselle E Lozano

**Affiliations:** Department of Entomology, University of California, Riverside, CA

**Keywords:** water availability, nutrition, pollinator, foraging, drought

## Abstract

Pollinators provide a key ecosystem service vital for the survival and stability of the biosphere. Identifying factors influencing the plant–pollinator mutualism and pollinator management is necessary for maintaining a healthy ecosystem. Since healthy beehives require substantial amounts of carbohydrates (nectar) and protein (pollen) from forage plants such as clover, we must assess how resources offered by plants change under limited water conditions in order to fully understand how drought modifies the pollination mutualism. Here we document how reduced water availability leads to decreased nectar quality and quantity and decreased protein quality of pollen. Furthermore, we provide conclusive evidence that these lower quality resources lead to decreased survival and productivity in both developing honey bees (Hymenoptera: Apidae) and bumble bees (Hymenoptera: Apidae). The results emphasize the importance of the nutritional effects of reduced water on bees when predicting shifts of pollination mutualisms under climate change.

Bees are some of the most economically important beneficial insects in the United States (James and Pitts-Singer 2008). Because of the vital ecological economic importance of their pollination services, maximizing bee health is of paramount importance. Stressors at the ecosystem-level can affect both plant reproduction and pollinator health (Cayan et al. 2008). One such emerging threat is climate change, which includes multiple stressors, such as warmer temperatures and increasing incidence of drought (Intergovernmental Panel on Climate Change 2002, Phillips et al. 2018). Shifts in temperature or precipitation can significantly alter plant physiology, phenology (Penuelas et al. 2004, Andresen et al. 2010, Brunet and Larson-Rabin 2012) and survival (see references in McDowell et al. 2013). Because bees are obligate florivores and acquire most of their nutrients from nectar and pollen (Jordano et al. 2006, Winfree 2010), any altered availability and quality of bee forage may affect bee health (Di Pasquale et al. 2013, Ruedenauer et al. 2015, Smart et al. 2019), with cascading effects on pollination services.

Diet affects bee development and health, particularly immunocompetency (Alaux et al. 2010), and bees require a rich and diverse diet for optimal health (Di Pasquale et al. 2013). Therefore, to investigate how reduced water conditions modify the pollination mutualism, we must first quantify how reduced water affects the resources offered by the plant, and second, measure how such changes in floral nutritive value affect bee health and productivity. Under severe drought stress or extreme desiccation or dehydration strain as defined by Blum and Tuberosa (2018), plants may abort flowers entirely (Peterson et al. 1993, Fang et al. 2010) or senesce (McDowell 2011). However, plants experiencing limited water conditions do not necessarily experience desiccation; rather, plants under low, but not dehydration-inducing conditions may produce fewer (Burkle and Runyon 2016, Phillips et al. 2018) or smaller flowers (Gallagher and Campbell 2017). Although, how the nutritive value of floral resources is influenced by reduced water has received little attention.

Changes in floral resource availability and quality bring about shifts in foraging behavior (Harder 1986) and floral selection (Cnaani et al. 2006). Recent studies found that variations in water available to plants led to altered pollinator visitation rates (Gallagher and Campbell 2017, Descamps et al. 2018), as well as detectable changes in nectar volume and corolla size (Gallagher and Campbell 2017, Descamps et al. 2018, Brunet and Van Etten 2019). Such changes in direct response to water availability may lead to a morphological mismatch between corolla length and pollinator tongue length (Gérard et al. 2020). While there have been studies documenting the negative effects of drought on nectar volume and sugars (e.g., Waser and Price 2016, Descamps et al. 2018), little is known about how water limitation affects the nutritive qualities and availability of *both* nectar and pollen. Furthermore, no studies to date directly link such reductions in floral rewards to decreases in bee development and productivity.

Here, we use a combination of manipulative experiments to follow and quantify the direct impacts of water limitation on the quantity and quality of floral rewards and to assess the subsequent impacts on bee development and colony productivity. We test the hypothesis that nutrition available to honey bees and bumble bees is reduced when plants have less water available yet are not experiencing water stress. Understanding the impacts of reduced water availability on pollen and nectar production and consequences for bee health will help develop recommendations to maximize pollination services and nutrient availability under restricted water or drought conditions.

## Materials and Methods

### Water Limitation Impacts on Nectar and Pollen

We measured the quantity and quality of floral nectar and pollen produced by florets under optimum and lower water conditions. For the experimental trials, we focused on a common forage plant native to western North America, tomcat clover (*Trifolium willdenovii*). Seeds were obtained from Larner Seeds (Bolinas, CA), scarified, and germinated in germination trays using UC Soil Mix III (Supp Table S1 [online only]). For each experiment we grew 160 plants in Deepots (D40, Stuewe & Sons) in a temperature-controlled greenhouse at the University of California, Riverside: 80 plants experienced optimal water conditions and 80 plants experienced reduced water conditions (drought treatment). All plants were watered using the greenhouse’s fertilized water system (Peters Excel 25-5-20 at 1:100 injection rate, Scotts Miracle-Gro Company, Marysville, OH). Optimal water conditions were assessed by determining how much water was required for the soil to be at field capacity (Colman 1947). The reduced water treatment was watered just as often as the optimal treatment but received 30% less water than the optimal treatment. This reduction corresponded with the median required water use reductions for the state of California (California Water Boards 2016), but is above the levels that typically induce drought stress or dehydration strain (Blum and Tuberosa 2018). We recorded the date of first bloom and the number and size of all inflorescences for all plants. We also collected nectar and pollen from these 160 plants.

To collect nectar and pollen, we removed entire inflorescences one day after the first florets opened, and we counted the number of open florets for each inflorescence. For each open floret, we used microscissors to separate the anthers from the filaments and set these aside for pollen collection (see below). Each floret was then pinned to a cork stopper (Kearns and Inouye 1993). This stopper was inverted into a 1.5 ml centrifuge tube and spun at 2500 × g for 4 min. Clover nectar was collected from an average of 73.1 ± 14.1 florets per plant and pooled for each plant over 20 d in pre-weighed tubes. Using micro-capillary tubes, the total volume of collected nectar was measured (Cruden and Hermann 1983) and divided by the sum of collected florets, yielding an average volume of nectar produced per floret per plant. The sugar concentration of each pooled nectar sample was then determined in %BRIX using a refractometer. For most nectar samples, we used a hand-held refractometer (Bellingham and Stanley, Eclipse 45–81); however, for the few nectar samples with very high %BRIX (>50%) we used a refractometer designed for high sugar content (Ade Advanced Optics, RHW-80ATC).

In addition to harvesting nectar from each plant during its bloom period, we collected the anthers for each floret (Supp Material [online only]). We subsequently separated the pollen from anthers using a microcentrifuge, and pooled pollen by plant in a pre-weighed microcentrifuge tube. We collected pollen from an average of 85.8 ± 22.6 florets per plant and weighed pollen using a Sartorius M2P balance. To assess pollen quantity, we divided the total mass of pollen collected per plant by the number of florets collected yielding the average mg of pollen per floret for each plant. To analyze pollen quality, we extracted total protein using a protocol adapted from Vanderplanck et al. (2014) and Wang et al. (2006) (Supp Material [online only]), then quantified total protein using a bicinchoninic acid (BCA) Protein Assay following manufacturer’s directions (Pierce, Thermo Scientific).

To determine if reduced water affected plant fitness, we grew an additional 80 plants (40 plants under each of the two watering conditions), hand self-pollinated the seven to 10 florets per inflorescence per plant using a camel-hair paintbrush and quantified the resulting seed set (e.g., number of seeds per floret and seed weight). Pollen and nectar collected from these 80 plants were included in the assessments of floral reward quantity and quality described above. At the time of seed collection, we harvested, dried, and weighed each plant to obtain total plant biomass.

### Water Limitation Impacts on Bee Survival Via Changes in Floral Resources

To link the impacts of reduced water availability to plants directly to bee survival and productivity, we developed artificial diets based on the nutritional composition offered by clover inflorescences. Using a sucrose/glucose/fructose assay kit (Megazyme Bray, Ireland), we found a 50:23:27 sucrose:glucose:fructose ratio in clover nectar in both optimally treated and reduced water treatments. Pilot experiments revealed that nectar from reduced water plants had 17% lower sugar concentration (BRIX) as compared to the nectar of optimum plants and that pollen from reduced water treatment had 10% less protein than pollen of optimum watered plants. Based on the differences in our nutritional analyses of clover described above, we manipulated the protein quality of bee diets by manipulating the amount of royal jelly (Crockett Honey, Tempe AZ) provided to honey bees or the pollen (Brushy Mountain, Moravian Farms, NC) provided to bumble bees. Quality of artificial diets were double checked using a refractometer for nectar and the assay kits described above for pollen to ensure the appropriate differences in quality for the two treatments. These diets were provided to developing larval bees (*Apis mellifera*), bumble bee microcolonies (*Bombus impatiens*) (Cnaani et al. 2002) or to bumble bee colonies (*B. impatiens*).

#### Effect of Nutrition on Developing Honey Bees

Following the methods of Di et al. (2016), Aupinel et al. (2005), and Peng et al. (1992), we obtained 1-d-old larvae from an Apis mellifera colony at the University of California, Riverside apiary. Using grafting tools (Sinova, Zhengzhou, China), we grafted 96 one-day-old honey bee larvae onto 250 µl of artificial diet in queen cell cups. Cell cups were then placed into 48-well plates (Corning, New York, NY), covered, and maintained in a dark environmental chamber (34 ± 0.1°C, 95 ± 0.5 % humidity). All larvae were assessed for four hours and 24 h after grafting to determine the rate of mortality due to grafting. Diets were modified from Di et al. (2016) to reflect our observed 10% and 17% reductions in protein and carbohydrates, respectively, as a result of water limitation to clover. The optimum water diet consisted of 53% (w/w) royal jelly, 6.5% sucrose, 3.1% glucose, 3.4% fructose, and 34% water, while the reduced water diet was comprised of 47.6% royal jelly, 5.4% sucrose, 2.6% glucose, 2.9% fructose, and 41.5% water. To reflect the reduced protein available to developing larvae, we altered the concentration of royal jelly in the larval provisions. Forty-eight larvae received the optimum diet and 48 larvae received a diet reflecting the relative carbohydrate and protein concentrations of the reduced water plants. Bees were observed between 0900 and 1000 hours every morning, noting growth, onset of pupation, adult emergence, and mortality. All adults were weighed at the end of the experiment.

#### Effect of Nutrition on Bumble Bees

In addition to honey bees, we raised Bombus impatiens on each of the two diets based off the nutritional values of the clover grown under the optimal or the reduced water conditions. We created and maintained 74 microcolonies each consisting of three workers from the same source colony, on these artificial diets (i.e., 37 microcolonies per treatment) using six source colonies. Small, queenless microcolonies are commonly used to study bumble bee development and survival (Cnaani et al. 2002, Moerman et al. 2017) and allows for treatments to be balanced across source colonies (Melgarejo et al. 2018). To reflect the 10% reduction in protein and 17% reduction in carbohydrates due to water reduction in clover, we created the following diets. The optimum water diet consisted of 0.250 g pollen and a sugar solution of 29.4% sucrose, 13.5% glucose, 15.9% fructose, and 41.2% water, while the reduced water diet included 0.225g pollen and a sugar solution of 24.8% sucrose, 11.4% glucose, 13.4% fructose, and 50.6% water. All bumble bee workers were marked with unique number tags to allow for individual identification and calculation of forager lifespans and mortality rates. Fresh food was provided daily. We also noted the creation of honey pots and any incidence of worker egg laying in the microcolonies.

We also raised entire Bombus impatiens colonies (*n* = 4 colonies total, two colonies per treatment) ad libitum on these artificial diets. At the beginning of the experiment, each colony had one queen and 40–50 workers. Similar to microcolonies, all workers were individually marked. We recorded worker body mass, survival and mortality, as well as new worker (i.e., callow) and gyne production daily.

### Statistical Analyses

All analyses were conducted in R v. 3.6.0 (R Core Team 2020) and means are reported ± standard error. All generalized linear mixed-effects models (GLMMs) were conducted using the lme4 package (Bates et al. 2013). Gaussian error distribution was used unless otherwise noted. All plant phenological variables (time from scarification to first floret, number of inflorescences, number of florets per inflorescence, total number of florets, total plant dry biomass, and seed weight) were analyzed using GLMMs where treatment was the fixed effect and random effects were plant ID and sample collection date. Plant productivity (seed set per inflorescence and the number of pollinated florets per inflorescence that set seed) was similarly assessed using a Poisson error structure. To assess the effects of water treatment on the quantity or quality of nectar or pollen on a per floret basis, we used GLMMs with treatment as the fixed effect, plant ID and sample collection date as random effects. To assess treatment effects on the quantity or sugar concentration of nectar and the quantity and protein concentration of pollen on a per plant basis, we used GLMMs with treatment and number of florets as fixed effects, sample collection date as a random effect.

For each bee species separately, survival curves were estimated for both diets using the Kaplan-Meier method and compared using the ‘survdiff’ function in package survival (Therneau 2015). For honey bees, we used χ ^2^ tests to compare number of bees reaching each stage of development and mortality before adulthood between treatments. In package stats (R Core Team 2020), we used *t*-tests to assess treatment effects on development time and body mass of bees. For *Bombus* microcolonies, a GLMM examined the effect of treatment on forager body mass with microcolony identity as a random effect. We calculated odds ratio (OR) and Pearson χ ^2^ for the creation of honey pots and incidence of egg laying across microcolonies by diet. For full bumble bee colonies, we used χ ^2^ tests to compare the total numbers of new workers and gynes produced between the treatments.

## Results

### Water Limitation Impacts on Nectar and Pollen

**Fig. 1.**
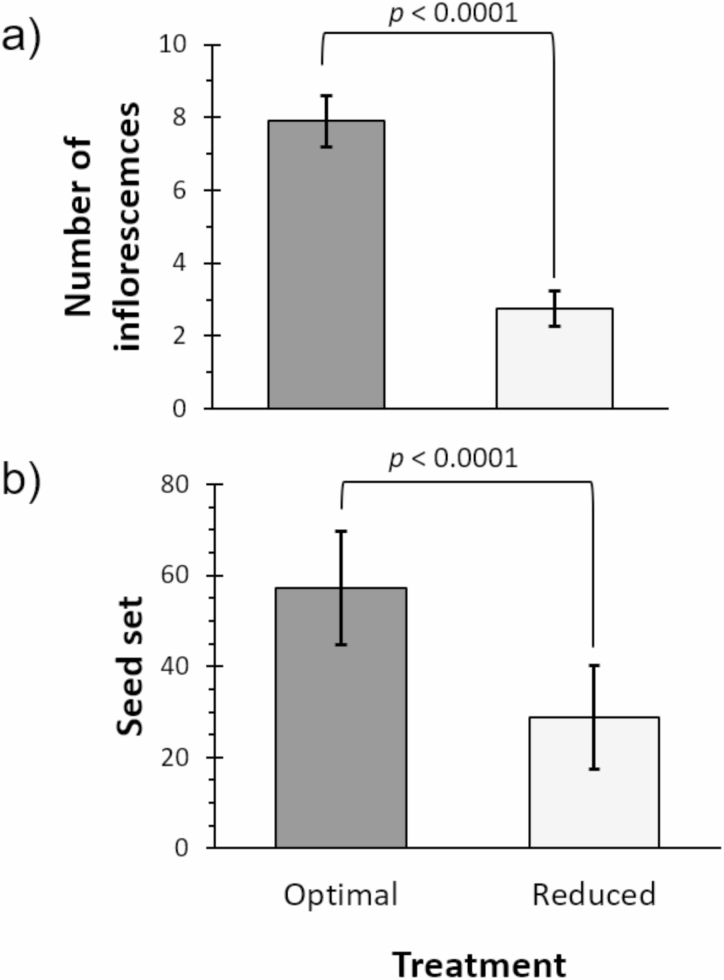
Reduced water impacts plant productivity. (a) Number of inflorescences per clover plant when grown under optimal and 30% reduced water conditions. (b) Number of seeds per clover plant produced under optimal and 30% reduced water conditions.

Watering regimes altered the phenology of clover and the quality of its floral rewards. Reduced water led to increased time until first floret opening by 3 d (*F*_1,71_ = 4.70, *P* = 0.034) and resulted in half as many inflorescences per plant as compared to the optimal water treatment (Fig. 1a: *F*_1,107_ = 73.72, *P* < 0.00001). Plants in the optimal treatment produced significantly more florets per inflorescence (12.0 ± 0.3 florets) than those in the reduced water treatment (9.9 ± 0.3 florets) (*F*_1,8_ = 18.45, *P* = 0.0023). Moreover, optimal plants produced an average of 165.4 ± 15.8 total florets, while reduced water plants had an average of 118.6 ± 5.8 total florets. Watering regime also had an effect on total plant biomass: plants in the optimal watering treatment had 46% more dry plant biomass than plants in the reduced water treatment (*F*_1,83_ = 19.36, *P* < 0.0001).

Watering treatment also affected seed set: the number of seeds produced per plant were more than twofold higher in the optimal treatment as compared to the reduced water treatment (Fig. 1b: *F*_1,8_ = 11.64, *P* = 0.0092). Across treatments, the percentage of florets per inflorescence that set seed were similar (reduced water: 48.6% and optimal: 50.9%); however, the total number of seeds produced per inflorescence did differ among treatments (*F*_1,651_ = 16.05, *P* < 0.0001), with optimal plants producing 23% more seeds per inflorescence (5.9 ± 0.3 seeds vs 4.7 ± 0.2 seeds, respectively). The weight of an individual seed, however, was unaffected by treatment (reduced water: 2.91 ± 0.2 mg and optimal: 3.04 ± 0.2 mg; *F*_1,9_ = 0.0072, *P* = 0.93).

#### Nectar Quantity and Quality

**Fig. 2.**
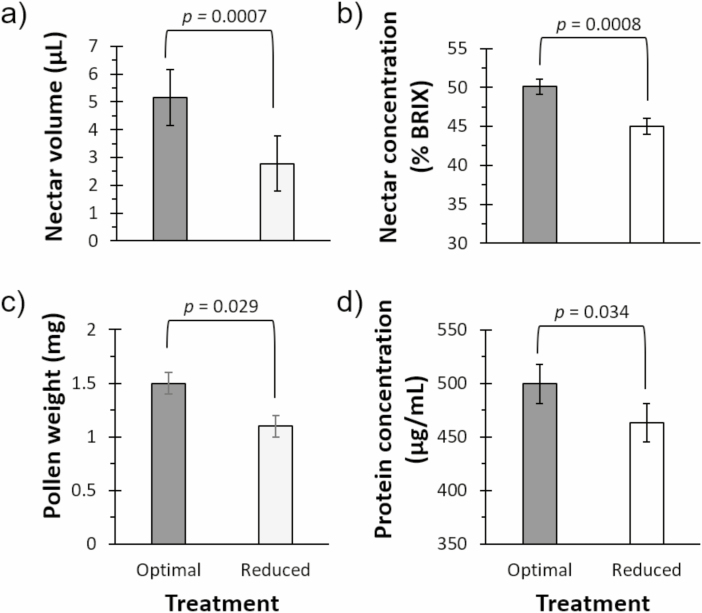
Reduced water treatment leads to decreases in clover floral nutrition measured at the plant level. Flower (a) nectar volume and (b) total sugar concentration both decreased with reduced water. (c) Pollen mass and (d) total protein concentration was also highest in the optimal water treatment.

Inflorescences from optimal water plants produced with an average of 0.09 ± 0.01 µl of nectar per floret while inflorescences from low water plants exhibited an average of 0.03 ± 0.01 µl of nectar per floret (*F*_1,125_ = 7.86, *P* = 0.0059). Consequently, we observed that higher water treatment led to increased nectar volumes overall as compared to the reduced water treatment (Fig. 2a: *F*_1,125_ = 12.02, *P* = 0.0007). Moreover, nectar from optimal plants exhibited 11% higher sugar concentration (BRIX) than nectar from reduced water plants (Fig. 2b: *F*_1,66_ = 12.38, *P* = 0.0008).

#### Pollen Quantity and Quality

The mass of pollen per floret did not differ between treatments: 0.076 ± 0.007 mg for optimal water plants and 0.085 ± 0.01 mg for reduced water plants (*F*_1,114_ = 1.27, *P* = 0.26). Importantly, the total amount of pollen produced per plant decreased 36% by the water treatment (Fig. 2c: *F*_1,114_ = 4.87, *P* = 0.029), reflecting the difference in total number of florets by treatment. Analysis of total raw protein for these pollen samples revealed 8% more protein in the optimal treatment than pollen from the reduced water treatment (Fig. 2d: *F*_1,28_ = 4.97, *P* = 0.034).

### Water Limitation Impacts on Bee Survival Via Changes in Floral Resources

#### Effect of Nutrition on Developing Honey Bees

**Fig. 3.**
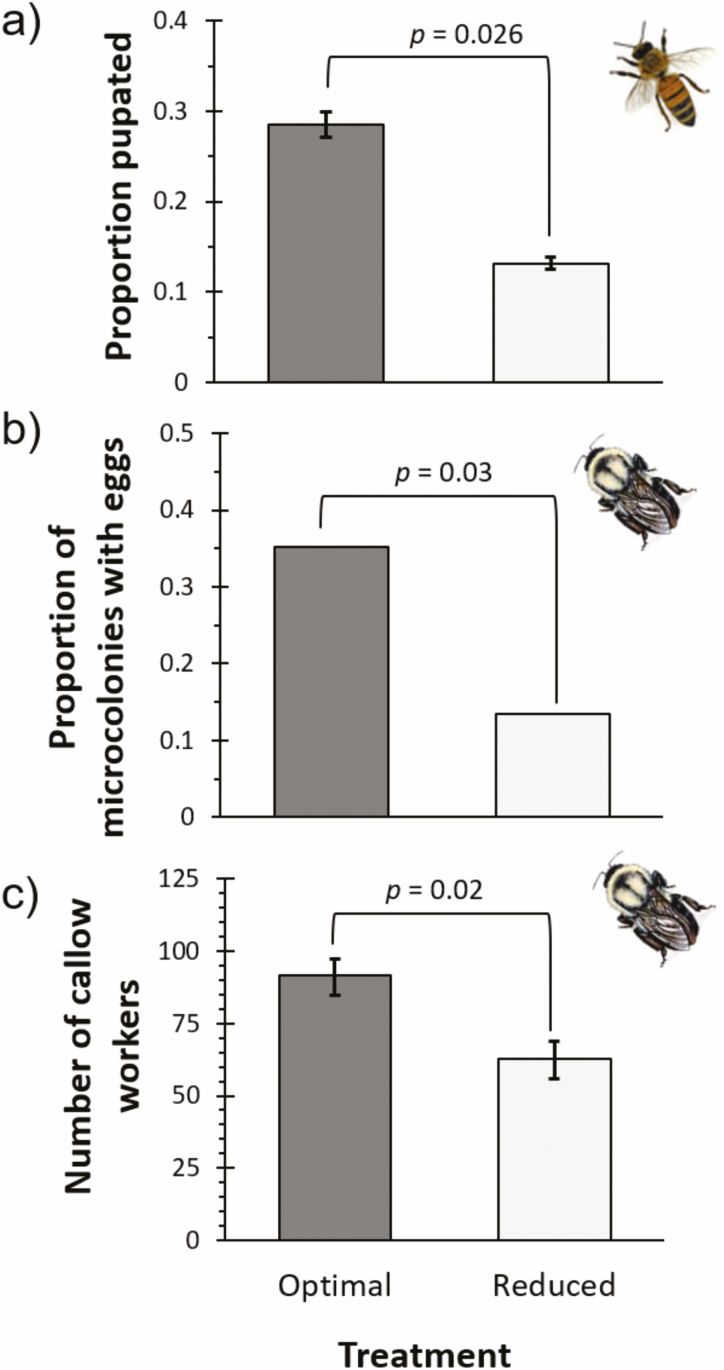
Reduced water negatively impacts honey bees and bumblebees. (a) Proportion of larval honey bees that successfully pupated when grafted onto diets based on clover under optimal and 30% reduced water conditions. (b) Proportion of *Bombus impatiens* microcolonies (*n* = 37 microcolonies per treatment) that laid eggs by diet treatment. (c) Number of new bumble bee workers (callows) produced in colonies raised on diets based on clover grown under optimal and 30% reduced water conditions.

Mortality due to the grafting process did not differ between the treatments (optimal: 19% vs reduced water: 15%). There was a significant effect of diet quality on honey bee survival post-grafting (Kaplan-Meier: X^2^ = 6.9, *P* = 0.009), where bees in the optimal diet treatment had 26% higher survivorship than those on the reduced water diet. Nutrition also had a significant impact on total development time from egg until adult emergence (*t*_13_ = 3.42, *P* = 0.0046). Individuals raised on the optimal diet emerged as adults on 7.4 ± 0.2 d after pupating, whereas those from the reduced water diet emerged as adults 8.6 ± 0.3 d after beginning pupation (*t*_13_ = 2.76, *P* = 0.016). Pupae were first observed on day 9 for the optimal diet and first observed on day 11 for the reduced water diet. More than twofold more larval bees pupated successfully when grafted on the diet based on optimal clover plants’ nutritional make-up compared to those grafted on the diet based on nutritional quality of plants grown under reduced water (Fig. 3a: *t*_13_ = 2.51, *P* = 0.026). Despite qualitative differences, there was no treatment effect on adult body mass (optimal: 103.6 ± 5.6 mg vs reduced water: 87.1 ± 12.5 mg; *t*_13_ = 1.14, *P* = 0.27).

#### Effect of Nutrition on Developing Bumble Bees

Bumble bee microcolonies exhibited higher survival on the optimal diet as compared to the reduced water diet (Kaplan-Meier: X^2^ = 8.6, *P* = 0.0003). Notably, foragers lived 60% longer when raised on the optimal diet: 53.9 ± 2.7 d versus 33.6 ± 2.5 d, respectively (*F*_1,76_ = 28.634, *P* < 0.0001). There was no difference in forager body mass between the treatments (optimal: 91.4 ± 3.8 mg; reduced water: 93.0 ± 4.0 mg). Honey pots were built by 26% of the microcolonies overall, where diet had no effect on whether or not honey pots were present (OR: 1.53, X^2^ = 0.64, *P* = 0.42). However, significantly more microcolonies on the optimal diet (13/37) laid eggs than did microcolonies on the lower diet (5/37) (Fig. 3b: OR = 3.47, X^2^ = 4.7, *P* = 0.03).

We observed similar results for the *B. impatiens* colonies; colonies raised on the optimal diet had significantly higher individual bee survival than colonies on the reduced diet (Kaplan-Meier: X^2^ = 22.6, *P* = 2.0 e^-6^). Bumble bee colonies raised ad libitum on the optimal diet produced 50% more workers (Fig. 3c: X^2^ = 11.0, *P* = 0.0009: 91.5 ± 5.9 bees vs 62.8 ± 6.8 bees) and 35% more gynes (X^2^ = 7.03, *P* = 0.008) than the colonies raised ad libitum on the reduced water diet.

## Discussion

This is the first study, to our knowledge, to explicitly link water reduction, plant floral resources and bee health via the impact of water on nectar and pollen. We show that the nutritional quality and availability of floral resources for honey bees and bumble bees are significantly decreased by reduced water availability to clover plants. We raised these two commercially important pollinator species on artificial diets determined from the water reduction experiments and found that both bee species experienced significantly lower survival. Bumble bees showed lower incidences of egg laying (microcolonies) and overall productivity (i.e., new bees produced in full colonies). Decreased survival of developing honey bees and of bumble bee foragers may have subsequent colony-level effects. Moreover, a smaller workforce may have cascading negative effects on a social insect colony’s ability to collect resources (Schmid-Hempel and Schmid-Hempel 1998), and in the case of honey bees, to produce honey (Woyke 1984). Resource and nutritional intake by bees affect their development and survival. Because of our reliance on bees and bee pollination services (Klein et al. 2007, Kremen et al. 2007), we need to consider how floral resources and the nutrition they provide may shift under mechanisms of global change, such as invasions (Harmon-Threatt and Kremen 2015) and drought (Phillips et al. 2018).

While our artificial diets are able to mimic key aspects of floral nutrition (e.g., relative proportion of sugars, sugar concentration, total protein), it is important to note that these artificial diets were not designed to assess changes in other nutrients. Recent studies have documented that changes in pollen lipids alter the ability of bees to learn (Arien et al. 2015, Arien et al. 2018) as well as their foraging behavior with subsequent impacts on mortality (Vaudo et al. 2016, Moerman et al. 2017). While temperature can influence the sugar concentration and viscosity of royal jelly (Saricaoglu et al. 2019), if and how water availability directly influences royal jelly quality or composition is unknown. Further research is needed to assess how water restriction affects additional nutrients, such as pollen lipids and free amino acids in nectar and the composition of royal jelly.

Despite limitations of an artificial diet, we link the cascading effects of water limitation to pollinator survivorship and productivity via reductions in nectar and pollen quality. Our study demonstrates that plant phenology, numbers of inflorescences and florets, and floral resource metrics were all affected by a 30% decrease in water. Floral resource phenology and resource availability are known to be factors influencing bee populations (Wray et al. 2014, Dicks et al. 2015) and determining bee fitness (Ogilvie and Forrest 2017). For *Osmia lignaria*, a solitary bee, increases in resource availability have been found to favor earlier emergence (Farzan and Yang 2018). Similarly, the size of pollen provisions and their quality influences adult body size in the subsocial, small carpenter bee, *Ceratina calcarata* (Lawson et al. 2016), and adult life span in honey bees (Schmidt et al. 1987, Di Pasquale et al. 2013). Bees, like all insects, are reliant on food provisions to complete development. In some bees, restricted access to food during development can prolong development time (Burkle and Irwin 2009) and decrease gyne production (Goulson et al. 2015, Rotheray et al. 2017). Thus, these experiments can contribute to a proactive approach to managing the indirect impacts of water limitation on bee survivorship and productivity.

Because clover plants altered the quality of both pollen and nectar in response to water limitation, we are unable to ascribe lower bee survival and productivity to decreased nutritive value of pollen, nectar, or both. Based on previous studies examining effects of various diets (Schmidt et al. 1987, Rotheray et al. 2017), we currently hypothesize both are critically important. Future experiments could manipulate resources independently and at multiple concentrations to elucidate, which relative combination of resources has the largest effect on solitary and social bee species. While our study provides key insights and links water availability with decreased bee survivorship, further research should examine how wild bees foraging behaviors respond to water-induced changes in floral resources.

It is important to note that the plants in this study were *not* experiencing drought stress, as defined by Blum and Tuberosa (2018). These were otherwise healthy-looking plants that received less than optimum water allotments; despite not being under drought stress, plants in the reduced water treatment took longer to develop, produced fewer florets and less floral resources, and this ultimately led to lower seed set as compared to the optimal water treatment. These results correspond to other studies that documented phenology shifts (Penuelas et al. 2004) and decreased floral displays (Burkle and Runyon 2016, Phillips et al. 2018) when water is decreased.

Any such decreases in floral abundance will likely affect bee foraging behavior. For example, both bumble bees and honey bees often prefer to forage on larger floral displays (Mitchell et al. 2004, Higginson et al. 2006, respectively). In fact, many insects respond to decreased nutrient quality by increasing resource foraging, including crickets (Srygley and Lorch 2013), ants (Christensen et al. 2010, Dussutour and Simpson 2012), and bees (Wright 1988, Pernal and Currie 2000, Pernal and Currie 2001). Our experimental methodologies prevented such compensatory feeding (or increased consumption of the lower quality food to obtain similar nutritive values) from occurring in honey bees and bumble bee micro colonies. For honey bees, this was achieved by grafting each larva onto a standardized volume of artificial diet. For *Bombus* microcolonies, this was limited by providing each microcolony with the same amount of food resources. We did not measure total resource collection in the full bumble colonies and thus cannot assess whether compensatory feeding occurred at the colony level. However, we found that bumble bees raised on the reduced water diets fared significantly worse in all metrics measured than bumble bees raised on the optimal diets, despite each colony having unrestricted access to the specified experimental diets. Moreover, most studies looking at compensatory feeding and shifts in foraging behavior have not been conducted under an environmental stressor that is experienced at the landscape level (such as water limitation). Therefore, future work should examine how bees may compensate for landscape-wide declines in nutrition as simulated in our experiments in order to better inform bee management strategies.

The effect of decreased nutrition provided by flowers under reduced water conditions (Phillips et al. 2018) will depend on how water reduction is distributed across the landscape. If a single species or patch experiences reduced water, bees will be able to move on to another patch or more rewarding species. This may not directly impact the bees’ fitness but may have negative impacts on the pollination rates of the plants experiencing reduced water. However, under current predictions of climate change, we anticipate landscape-level water reduction (McDowell et al. 2013). This may take the form of reduced watering in managed landscapes due to regulated water restrictions (Mini et al. 2014, Huntsinger et al. 2017), or of natural drought conditions in natural ecosystems.

We document the cascading effects of limited water and its potential impact on bees. While this study focused on social bee species, solitary taxa are likely similarly affected. Since the size of offspring of solitary bees are determined by the quality and quantity of nectar and pollen in provisioning masses (Stone 1994), any landscape-wide decrease in nutritive value of floral resources will impact solitary bee survivorship and productivity as well. Our findings suggest that widespread reduced water, either due to natural weather patterns or mandatory water restrictions, may have a significant impact on the phenology, floral display, and nectar resources of bee forage. In short, the nutritive quality of flowers will need to be considered when developing strategies for promoting healthy bee populations under future climate conditions.

## Data Availability Statement

Data from this study are available from the Dryad Digital Repository: https://doi.org/10.6086/D14X10 (Wilson Rankin 2020).

## Supplementary Material

ieaa114_suppl_Supplementary_Material

## References

[CIT0001] Alaux C, Ducloz F, Crauser D, and Le Conte Y. 2010 Diet effects on honeybee immunocompetence. Biol. Lett. 6: 562–565. 20089536 10.1098/rsbl.2009.0986 PMC2936196

[CIT0002] Andresen L C, Michelsen A, Jonasson S, Schmidt I K, Mikkelsen T N, Ambus P, and Beier C. 2010 Plant nutrient mobilization in temperate heathland responds to elevated CO_2_, temperature and drought. Plant Soil. 328: 381–396.

[CIT0003] Arien Y, Dag A, Zarchin S, Masci T, and Shafir S. 2015 Omega-3 deficiency impairs honey bee learning. Proc. Natl. Acad. Sci. U. S. A. 112: 15761–15766. 26644556 10.1073/pnas.1517375112 PMC4697434

[CIT0004] Arien Y, Dag A, and Shafir S. 2018 Omega-6:3 ratio more than absolute lipid level in diet affects associative learning in honey bees. Front. Psychol. 9: e1001. 10.3389/fpsyg.2018.01001 PMC6018467 29971031

[CIT0005] Aupinel P, Fortini D, Dufour H, Tasei J, Michaud B, Odoux J, and Pham-Delegue M. 2005 Improvement of artificial feeding in a standard in vitro method for rearing Apis mellifera larvae. Bull. Insectology 58: 107.

[CIT0006] Bates D, Maechler M, and Bolker B. 2013 lme4: Linear mixed-effects models using S4 classes. http://CRAN.R-project.org/package=lme4

[CIT0007] Blum A, and Tuberosa R. 2018 Dehydration survival of crop plants and its measurement. J. Exp. Bot. 69: 975–981. 29325054 10.1093/jxb/erx445 PMC6018961

[CIT0008] Brunet J, and Van Etten M L. 2019 The response of floral traits associated with pollinator attraction to environmental changes expected under anthropogenic climate change in high-altitude habitats. Int. J. Plant Sci. 180: 954–964.

[CIT0009] Brunet J, and Larson-Rabin Z. 2012 The response of flowering time to global warming in an alpine plant: the impact of genetics and the environment. Botany 90: 319–326.

[CIT0010] Burkle L A, and Runyon J B. 2016 Drought and leaf herbivory influence floral volatiles and pollinator attraction. Glob. Chang. Biol. 22: 1644–1654. 26546275 10.1111/gcb.13149

[CIT0011] Burkle L, and Irwin R. 2009 The importance of interannual variation and bottom-up nitrogen enrichment for plant-pollinator networks. Oikos. 118: 1816–1829.

[CIT0012] California Water Boards 2016 Urban Water Supplier Conservation Standard for Extended Emergency Regulation Rulemaking. http://www.waterboards.ca.gov/water_issues/programs/conservation_portal/docs/emergency_reg/supplier_standards_effective030116.pdf

[CIT0013] Cayan D R, Maurer E P, Dettinger M D, Tyree M, and Hayhoe K. 2008 Climate change scenarios for the California region. Clim. Chang. 87: 21–42.

[CIT0014] Christensen K L, Gallacher A P, Martin L, Tong D, and Elgar M A. 2010 Nutrient compensatory foraging in a free-living social insect. Naturwissenschaften. 97: 941–944. 20689904 10.1007/s00114-010-0705-8

[CIT0015] Cnaani J, Schmid-Hempel R, and Schmidt J O. 2002 Colony development, larval development and worker reproduction in *Bombus impatiens* Cresson. Insectes Soc. 49: 164–170.

[CIT0016] Cnaani J, Thomson J D, and Papaj D R. 2006 Flower choice and learning in foraging bumblebees: effects of variation in nectar volume and concentration. Ethology 112: 278–285.

[CIT0017] Colman E A 1947 A laboratory procedure for determining the field capacity of soils. Soil Sci. 63: 277–283.

[CIT0018] Cruden R W, and Hermann S M. 1983 Studying nectar? Some observations on the art, pp. 223–241. *In* Bentley B and Elias T (eds.), The biology of nectaries. Columbia University Press, New York, NY.

[CIT0019] Descamps C, Quinet M, Baijot A, and Jacquemart A L. 2018 Temperature and water stress affect plant-pollinator interactions in *Borago officinalis* (Boraginaceae). Ecol. Evol. 8: 3443–3456. 29607037 10.1002/ece3.3914 PMC5869376

[CIT0020] Di N, Hladun K R, Zhang K, Liu T X, and Trumble J T. 2016 Laboratory bioassays on the impact of cadmium, copper and lead on the development and survival of honeybee (*Apis mellifera* L.) larvae and foragers. Chemosphere. 152: 530–538. 27011322 10.1016/j.chemosphere.2016.03.033

[CIT0021] Di Pasquale G, Salignon M, Le Conte Y, Belzunces L P, Decourtye A, Kretzschmar A, Suchail S, Brunet J L, and Alaux C. 2013 Influence of pollen nutrition on honey bee health: do pollen quality and diversity matter? PLoS One 8: e72016. 23940803 10.1371/journal.pone.0072016 PMC3733843

[CIT0022] Dicks L V, Baude M, Roberts S P, Phillips J, Green M, and Carvell C. 2015 How much flower-rich habitat is enough for wild pollinators? Answering a key policy question with incomplete knowledge. Ecol. Entomol. 40: 22–35. 26877581 10.1111/een.12226 PMC4737402

[CIT0023] Dussutour A, and Simpson S J. 2012 Ant workers die young and colonies collapse when fed a high-protein diet. Proc. Biol. Sci. 279: 2402–2408. 22357267 10.1098/rspb.2012.0051 PMC3350685

[CIT0024] Fang X, Turner N C, Yan G, Li F, and Siddique K H. 2010 Flower numbers, pod production, pollen viability, and pistil function are reduced and flower and pod abortion increased in chickpea (*Cicer arietinum* L.) under terminal drought. J. Exp. Bot. 61: 335–345. 19854801 10.1093/jxb/erp307 PMC2803204

[CIT0025] Farzan S, and Yang L H. 2018 Experimental shifts in phenology affect fitness, foraging, and parasitism in a native solitary bee. Ecology 99: 2187–2195. 30066397 10.1002/ecy.2475

[CIT0026] Gallagher M K, and Campbell D R. 2017 Shifts in water availability mediate plant-pollinator interactions. New Phytol. 215: 792–802. 28517023 10.1111/nph.14602

[CIT0027] Gérard M, Vanderplanck M, Wood T, and Michez D. 2020 Global warming and plant–pollinator mismatches. Emerg. Top. Life Sci. 4: 77–86. 32558904 10.1042/ETLS20190139 PMC7326340

[CIT0028] Goulson D, Nicholls E, Botías C, and Rotheray E L. 2015 Bee declines driven by combined stress from parasites, pesticides, and lack of flowers. Science. 347: e1255957. 10.1126/science.1255957 25721506

[CIT0029] Harder L D 1986 Effects of nectar concentration and flower depth on flower handling efficiency of bumble bees. Oecologia. 69: 309–315. 28311376 10.1007/BF00377639

[CIT0030] Harmon-Threatt A N, and Kremen C. 2015 Bumble bees selectively use native and exotic species to maintain nutritional intake across highly variable and invaded local floral resource pools. Ecol. Entomol. 40: 471–478.

[CIT0031] Higginson A D, Gilbert F S, and Barnard C J. 2006 Morphological correlates of nectar production used by honeybees. Ecol. Entomol. 31: 269–276.

[CIT0032] Huntsinger L, Hruska T V, Oviedo J L, Shapero M W K, Nader G A, Ingram R S, and Beissinger S R. 2017 Save water or save wildlife? Water use and conservation in the central Sierran foothill oak woodlands of California, USA. Ecol. Soc. 22: e12.

[CIT0033] Intergovernmental Panel on Climate Change 2002 Climate change and biodiversity. IPCC Technical Paper 5: 1–86.

[CIT0034] James R, and Pitts-Singer T L (eds.). 2008 Bee pollination in agricultural ecosystems. Oxford University Press, New York, NY.

[CIT0035] Jordano P, Bascompte J, and Olesen J M. 2006 The ecological consequences of complex topology and nested structure in pollination webs, pp. 173–199. *In* Waser N M and Ollerton J (eds.), Plant-Pollinator Interactions: From Specialization to Generalization. University of Chicago Press, Chicago, IL.

[CIT0036] Kearns C A, and Inouye D W. 1993 Techniques for pollination biologists. University Press of Colorado, Niwot, CO.

[CIT0037] Klein A M, Vaissière B E, Cane J H, Steffan-Dewenter I, Cunningham S A, Kremen C, and Tscharntke T. 2007 Importance of pollinators in changing landscapes for world crops. Proc. Biol. Sci. 274: 303–313. 17164193 10.1098/rspb.2006.3721 PMC1702377

[CIT0038] Kremen C, Williams N M, Aizen M A, Gemmill-Herren B, LeBuhn G, Minckley R, Packer L, Potts S G, Roulston T, Steffan-Dewenter I, et al 2007 Pollination and other ecosystem services produced by mobile organisms: a conceptual framework for the effects of land-use change. Ecol. Lett. 10: 299–314. 17355569 10.1111/j.1461-0248.2007.01018.x

[CIT0039] Lawson S P, Ciaccio K N, and Rehan S M. 2016 Maternal manipulation of pollen provisions affects worker production in a small carpenter bee. Behav. Ecol. Sociobiol. 70: 1891–1900.

[CIT0040] McDowell N G 2011 Mechanisms linking drought, hydraulics, carbon metabolism, and vegetation mortality. Plant Physiol. 155: 1051–1059. 21239620 10.1104/pp.110.170704 PMC3046567

[CIT0041] McDowell N G, Fisher R A, Xu C, Domec J C, Hölttä T, Mackay D S, Sperry J S, Boutz A, Dickman L, Gehres N, et al 2013 Evaluating theories of drought-induced vegetation mortality using a multimodel-experiment framework. New Phytol. 200: 304–321. 24004027 10.1111/nph.12465

[CIT0042] Melgarejo V, Wilson Rankin E E, and Loope K J. 2018 Do queen cuticular hydrocarbons inhibit worker reproduction in *Bombus impatiens*? Insectes Soc. 65: 601–608.

[CIT0043] Mini C, Hogue T S, and Pincetl S. 2014 Estimation of residential outdoor water use in Los Angeles, California. Landsc. Urban Plan. 127: 124–135.

[CIT0044] Mitchell R J, Karron J D, Holmquist K G, and Bell J M. 2004 The influence of *Mimulus ringens* floral display size on pollinator visitation patterns. Funct. Ecol. 18: 116–124.

[CIT0045] Moerman R, Vanderplanck M, Fournier D, Jacquemart A-L, and Michez D. 2017 Pollen nutrients better explain bumblebee colony development than pollen diversity. Insect Conserv. Divers. 10: 171–179.

[CIT0046] Ogilvie J E, and Forrest J R K. 2017 Interactions between bee foraging and floral resource phenology shape bee populations and communities. Curr. Opin. Insec. Sci. 21: 75–82. 10.1016/j.cois.2017.05.015 28822493

[CIT0047] Peng Y-S C, Mussen E, Fong A, Montague M A, and Tyler T. 1992 Effects of chlortetracycline of honey bee worker larvae reared in vitro. J. Invertebr. Pathol. 60: 127–133.

[CIT0048] Penuelas J, Gordon C, Llorens L, Nielsen T, Tietema A, Beier C, Bruna P, Emmett B, Estiarte M, and Gorissen A. 2004 Nonintrusive field experiments show different plant responses to warming and drought among sites, seasons, and species in a north-south European gradient. Ecosystems. 7: 598–612.

[CIT0049] Pernal S F, and Currie R W. 2000 Pollen quality of fresh and 1-year-old single pollen diets for worker honey bees (*Apis mellifera* L.). Apidologie 31: 387–409.

[CIT0050] Pernal S F, and Currie R W. 2001 The influence of pollen quality on foraging behavior in honeybees (*Apis mellifera* L.). Behav. Ecol. Sociobiol. 51: 53–68.

[CIT0051] Peterson G M, Westgate M E, and Peterson C M. 1993 Flower and pod development in water-deficient soybeans (*Glycine max* L. Merr.). J. Exp. Bot. 44: 109–117.

[CIT0052] Phillips B B, Shaw R F, Holland M J, Fry E L, Bardgett R D, Bullock J M, and Osborne J L. 2018 Drought reduces floral resources for pollinators. Glob. Chang. Biol. 24: 3226–3235. 29652102 10.1111/gcb.14130

[CIT0053] R Core Team 2020 R: a language and environment for statistical computing. http://www.R-project.org/

[CIT0054] Rotheray E L, Osborne J L, and Goulson D. 2017 Quantifying the food requirements and effects of food stress on bumble bee colony development. J. Apic. Res. 56: 288–299.

[CIT0055] Ruedenauer F A, Spaethe J, and Leonhardt S D. 2015 How to know which food is good for you: bumblebees use taste to discriminate between different concentrations of food differing in nutrient content. J. Exp. Biol. 218: 2233–2240. 26202778 10.1242/jeb.118554

[CIT0056] Saricaoglu F T, Cinar A, Demircan H, and Oral R A. 2019 Rheological and microstructural characterization of royal jelly at different temperatures. J. Food Process Eng 42: 11.

[CIT0057] Schmid-Hempel R, and Schmid-Hempel P. 1998 Colony performance and immunocompetence of a social insect, *Bombus terrestris*, in poor and variable environments. Funct. Ecol. 12: 22–30.

[CIT0058] Schmidt J O, Thoenes S C, and Levin M D. 1987 Survival of honey-bees, *Apis-mellifera* (Hymenoptera, Apidae), fed various pollen sources. Ann. Entomol. Soc. Am. 80: 176–183.

[CIT0059] Smart M D, Otto C R V, and Lundgren J G. 2019 Nutritional status of honey bee (*Apis mellifera* L.) workers across an agricultural land-use gradient. Sci. Rep. 9: e16252. 10.1038/s41598-019-52485-y PMC6838345 31700140

[CIT0060] Srygley R B, and Lorch P D. 2013 Coping with uncertainty: nutrient deficiencies motivate insect migration at a cost to immunity. Integr. Comp. Biol. 53: 1002–1013. 23670631 10.1093/icb/ict047

[CIT0061] Stone G N 1994 Activity patterns of females of the solitary bee *Anthophora plumipes* in relation to temperature, nectar supplies and body-size. Ecol. Entomol. 19: 177–189.

[CIT0062] Therneau T M 2015 A Package for Survival Analysis in S. version 2.38. https://CRAN.R-project.org/package=survival.

[CIT0063] Vanderplanck M, Leroy B, Wathelet B, Wattiez R, and Michez D. 2014 Standardized protocol to evaluate pollen polypeptides as bee food source. Apidologie. 45: 192–204.

[CIT0064] Vaudo A D, Stabler D, Patch H M, Tooker J F, Grozinger C M, and Wright G A. 2016 Bumble bees regulate their intake of essential protein and lipid pollen macronutrients. J. Exp. Biol. 219: 3962–3970. 27742891 10.1242/jeb.140772

[CIT0065] Wang W, Vignani R, Scali M, and Cresti M. 2006 A universal and rapid protocol for protein extraction from recalcitrant plant tissues for proteomic analysis. Electrophoresis. 27: 2782–2786. 16732618 10.1002/elps.200500722

[CIT0066] Waser N M, and Price M V. 2016 Drought, pollen and nectar availability, and pollination success. Ecology 97: 1400–1409. 27459771 10.1890/15-1423.1

[CIT0067] Wilson Rankin E E, Barney S K, and Lozano G E. 2020 Data from: reduced water negatively impacts social bee survival and productivity via shifts in floral nutrition. Dryad Digital Repository. doi:10.6086/D14X10. PMC7583269 33021636

[CIT0068] Winfree R 2010 The conservation and restoration of wild bees. Ann. N. Y. Acad. Sci. 1195: 169–197. 20536823 10.1111/j.1749-6632.2010.05449.x

[CIT0069] Woyke J 1984 Correlations and interactions between population, length of worker life and honey production by honeybees in a temperate region. J. Apic. Res. 23: 148–156.

[CIT0070] Wray J C, Neame L A, and Elle E. 2014 Floral resources, body size, and surrounding landscape influence bee community assemblages in oak-savannah fragments. Ecol. Entomol. 39: 83–93.

[CIT0071] Wright D H 1988 Temporal changes in nectar availability and *Bombus appositus* (Hymenoptera: Apidae) foraging profits. Southwest. Nat. 33: 219–227.

