# Supplementary material for: Reduced Water Negatively Impacts Social Bee Survival and Productivity Via Shifts in Floral Nutrition
Source: J Insect Sci. 2020 Oct 6;20(5):15. doi: 10.1093/jisesa/ieaa114 (PMC7583269; doi:10.1093/jisesa/ieaa114)
Supplement: ieaa114_suppl_Supplementary_Material [file ieaa114_suppl_supplementary_material.docx]

*Supporting Information*

**Reduced water negatively impacts social bee survival and productivity via shifts in floral nutrition**

Erin E. Wilson Rankin^1*^, Sarah K Barney^1^ and Giselle E. Lozano^1^

^1^ Department of Entomology, University of California, Riverside, 900 University Ave, Riverside CA 92521

^*^ corresponding author: [e.wilson.rankin@gmail.com](mailto:e.wilson.rankin@gmail.com), ORCID ID: 0000-0001-7741-113X

**Table S1. Standard UC Soil Mix III Recipe from the Department of Agricultural Operations at UC Riverside. For more details, visit http://agops.ucr.edu/soil/.**

| **Ingredient** | **Per Cubic Meter of Soil** |
| --- | --- |
| Plaster sand | 0.439 m^3^ |
| Peat moss | 0.326 m^3^ |
| Potassium nitrate (KNO_3_) | 0.11 kg |
| Limestone flour | 0.68 kg |
| Phosphate | 0.57 kg |
| Dolomite | 1.70 kg |
| Magnesium | 0.032 kg |
| Iron | 0.059 kg |
| Manganese | 0.024 kg |
| Zinc | 0.023 kg |
| Copper | 0.050 kg |

**Supplementary methods: Detailed methods for nectar and pollen collection from clover inflorescences**

Using microscissors, we removed only the open florets from each plant by cutting the small green stem holding each floret onto the inflorescence. For each floret, we used microscissors to snip the banner to the top of the calyx. Then the microscissors were used to lightly push at the bottom center of the keel and slide up. If done slowly and carefully, this will expose the anthers covered in yellow pollen. We then cut off the anthers with the shortest amount of filament as possible. Anthers were stored in a microcentrifuge tube, centrifuged at max speed to separate the pollen from the anthers. We then pooled pollen samples across florets for each plant, and assessed the total protein available in the pollen sample on a per plant basis.

After anthers were carefully removed, we cut away the rest of the petals down to the top of the calyx. Each floret was then pinned to the larger side of a small cork, such that the top of the calyx is facing towards the pin head. We repeated the process for every floret for that plant. After turning the cork upside-down in a microcentrifuge tube, we then centrifuged the florets for 4 minutes at 2500 x g to collect the nectar. Nectar samples were pooled across all florets for each plant for further analysis.

**Supplementary methods: Modification of Vanderplank et al. (2014) and Wang et al. (2006)**

**Protein Content Analysis**

**Sample Preparation**

1. Weigh pollen and combine until each sample tube (2 mL) has 5 mg pollen. Flash freeze.
2. Adding enough acetone to cover, bead beat with equivalent volume of glass beads. Repeat up to five times. Remove supernatant and add to new 2 mL tube.

**Protein Precipitation and Contaminant Removal (all washes chilled first)**

1. Add 1 mL 10% Trichloroacetic acid (TCA)/acetone; vortex vigorously; centrifuge at 16000g for 3 min (4 degrees C); discard supernatant.
2. Add 1 mL 80% methanol with 0.1 M ammonium acetate; vortex vigorously; centrifuge at 16000g for 3 min (4 degrees C); discard supernatant.
3. Add 1 mL 80% acetone; vortex vigorously; centrifuge at 16000g for 3 min (4 degrees C); discard supernatant.

**Acetone Residual Removal**

1. Incubate at 50C for at least ten minutes (air drying may be sufficient)
2. Add 2-mercaptoethanol and Sodium dodecyl sulfate (SDS) to SDS buffer while waiting

**Extraction of Polypeptides**

1. Add 500 uL SDS buffer; vortex *vigorously* and incubation time to 10 minutes at 4C
2. Add 500 uL Tris-buffered phenol. Vortex and incubate for 10 minutes at RT in heater/shaker
3. Centrifuge at 16000 g for 10 min at 4C.
4. Recover 400 uL of upper phenol phase and transfer to new 2 mL tube.

**Precipitation of Polypeptides + Wash/Dry Pellet**

1. Add 1 mL 0.1 M ammonium acetate methanolic solution and incubate at -20C overnight
2. NEXT DAY: centrifuge at 16000 g for 10 min (4C) and discard supernatant
3. Wash pellet with icecold 100% methanol, vortex and centrifuge at 16000 for 10 min (4C)
4. Wash pellet with icecold 80% acetone, vortex and centrifuge at 16000 for 10 min (4C)
5. Allow polypeptides to air dry briefly
6. Dissolve in 4M guanidine HCl

**References**

Vanderplanck, M., Leroy, B., Wathelet, B., Wattiez, R. and Michez, D. (2014) Standardized protocol to evaluate pollen polypeptides as bee food source. *Apidologie,* **45**, 192-204.

Wang, W., Vignani, R., Scali, M. and Cresti, M. (2006) A universal and rapid protocol for protein extraction from recalcitrant plant tissues for proteomic analysis. *Electrophoresis,* **27**, 2782-2786.
